# Supplementary material for: Fluorescence Super-Resolution Imaging Chip for Gene Silencing Exosomes
Source: Sensors (Basel). 2023 Dec 28;24(1):173. doi: 10.3390/s24010173 (PMC10781284; doi:10.3390/s24010173)
Supplement: Supplementary file 1 [file sensors-24-00173-s001.zip › sensors-2709291-supplementary.pdf]

# Fluorescence Super-Resolution Imaging Chip for Gene Silencing Exosomes

Gaoqiang Yin, Tongsheng Qi, Jinxiu Wei, TingYu Wang, Zhuyuan Wang, Yiping Cui, Shenfei Zong *

1 Southeast University, School of Electronic Science & Engineering, Advanced Photonics Center, Nanjing 210096, China

* sfzong@seu.edu.cn;

***Supplementary Table S1. The sequences of docking and imager strands for individual biomarker.***

| Identification | Sequences |
|---|---|
| Docking strands for PD-L1 | 5'-Azide-TTATCTACATA-3' |
| Imager strands for PD-L1 | 5'-TATGTAGATC-Cy5-3' |
| CD63 adaptor strands | 5'-CAC CCC ACC TCG CTC CCG TGA CAC TAA TGC TA- $(CH_2)_6$-$NH_2$-3' |
| PD-L1 adaptor strands | 5'-Alexa Fluor 647-T ACA GGT TCT GGG GGG TGG GTG GGG AAC CTG TT-3' |
| PD-L1 siRNA sense strand | 5'-GGAUAAGAACAUUAUUCAA[dT][dT]-3' |
| PD-L1 siRNA antisense strand | 5'-UUGAAUAAUGUUCUUAUCC[dT][dT]-3' |

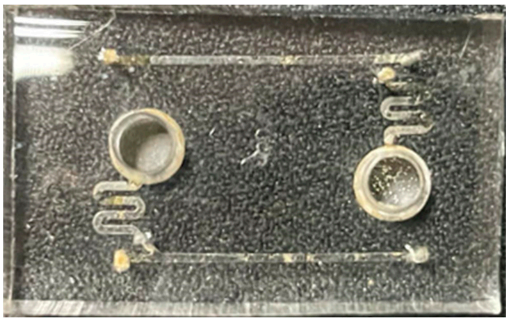

**Figure S1. The photograph of the microfluidic chip.**

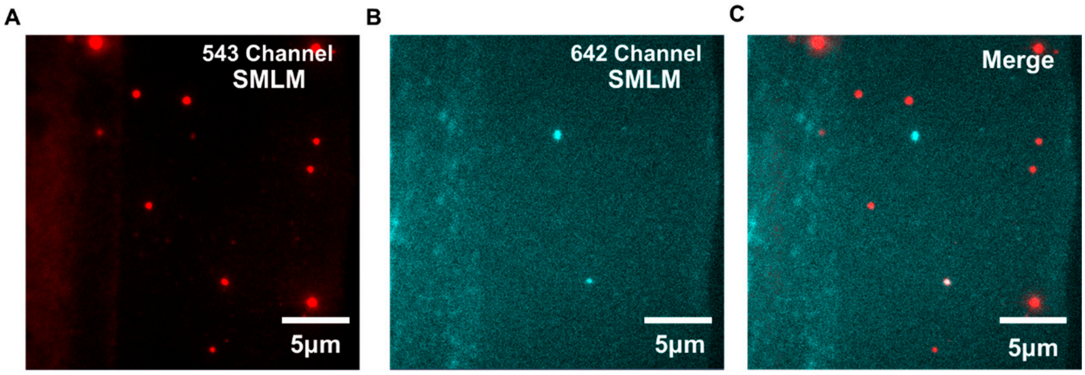


**Figure S2. Investigating the specificity of PD-L1 aptamers toward exosomal PD-L1 using nonspecific control probes.** (A) PKH26 membrane dye channel (exosomes), (B) Alexa fluor 647

channel (control probes), (C) merged image of the two channels.
